# Redefining Psychiatry Placements: Improving Medical Education Through the UTLA Scheme

**DOI:** 10.1192/bjo.2025.10293

**Published:** 2025-06-20

**Authors:** Sanaa Moledina, Hetal Acharya

**Affiliations:** Northamptonshire Healthcare NHS Foundation Trust, Northampton, United Kingdom

## Abstract

**Aims:** Psychiatry placements can be challenging for medical students due to unpredictable settings, emotionally charged patient interactions, and potential risks. Limited hands-on experience, inadequate supervision, inconsistent teaching across the wards, and poor induction further amplify students’ apprehensions, reduce engagement, and deter them from considering a career in psychiatry. To address these challenges, the University of Leicester Medical School (LMS) initiated the Undergraduate Teaching Liaison Associates (UTLA) scheme within Northamptonshire Healthcare NHS Foundation Trust to improve medical education in the mental health sector. This programme, grounded in the Near-Peer Teaching model, provides structured mentorship and personalized guidance to medical students. The quality improvement project focused on implementing and adapting the UTLA initiative specifically for psychiatry placements.

**Methods:** The project introduced the UTLA Blueprint, adapted from the LMS UTLA Handbook, to provide a structured yet flexible framework for guiding UTLAs in supporting students’ needs. Key UTLA responsibilities included conducting ward orientation, facilitating learning outcomes, supporting portfolio development, and performing regular well-being check-ins. During induction, Medical Student Handbook and UTLA flyers were distributed, and Meet and Greet sessions were organized. A UTLA network was also established to foster collaboration and shared learning. Data collection involved student surveys and UTLA interviews to evaluate the initiative’s impact.

**Results:** Eleven medical students participated in the survey, with 73% rating the UTLA programme eight or higher on the satisfaction scale, indicating high overall satisfaction. Furthermore, 64% of respondents found UTLA support highly accessible, and all expressed willingness to recommend it to their peers. The most frequently cited areas of UTLA assistance included arranging learning opportunities, providing well-being check-ins, and facilitating case-based discussions. The well-being check-ins and the Medical Student Handbook were distinguished as the two most useful elements. Suggested areas for improvement included clarifying UTLA roles at the start of placements, optimizing the frequency of check-ins to balance support and avoid over-contact (with 73% of students preferring 2–3 check-ins per fortnight), and educating UTLAs about required placement competencies. The UTLAs reported gaining valuable insights from frequent meetings, which facilitated shared learning and encouraged creative approaches to support.

**Conclusion:** The UTLA initiative provides a personalized mentorship and supervision framework that enables medical students to excel during psychiatry placements and fosters interest in psychiatry as a career. Future steps include improving communication about UTLA roles, adjusting check-in frequency, and conducting regular evaluations to refine the scheme based on feedback from both students and the UTLAs.

